# Evaluation of the Traditional and Revised WHO Classifications of Dengue Disease Severity

**DOI:** 10.1371/journal.pntd.0001397

**Published:** 2011-11-08

**Authors:** Federico Narvaez, Gamaliel Gutierrez, Maria Angeles Pérez, Douglas Elizondo, Andrea Nuñez, Angel Balmaseda, Eva Harris

**Affiliations:** 1 Infectious Diseases Unit, Hospital Infantil Manuel de Jesús Rivera, Managua, Nicaragua; 2 Sustainable Sciences Institute, Managua, Nicaragua; 3 National Virology Laboratory, Centro Nacional de Diagnóstico y Referencia, Ministry of Health, Managua, Nicaragua; 4 Division of Infectious Diseases and Vaccinology, School of Public Health, University of California, Berkeley, California, United States of America; Institute of Tropical Medicine (NEKKEN), Japan

## Abstract

Dengue is a major public health problem worldwide and continues to increase in incidence. Dengue virus (DENV) infection leads to a range of outcomes, including subclinical infection, undifferentiated febrile illness, Dengue Fever (DF), life-threatening syndromes with fluid loss and hypotensive shock, or other severe manifestations such as bleeding and organ failure. The long-standing World Health Organization (WHO) dengue classification and management scheme was recently revised, replacing DF, Dengue Hemorrhagic Fever (DHF), and Dengue Shock Syndrome (DSS) with Dengue without Warning Signs, Dengue with Warning Signs (abdominal pain, persistent vomiting, fluid accumulation, mucosal bleeding, lethargy, liver enlargement, increasing hematocrit with decreasing platelets) and Severe Dengue (SD; dengue with severe plasma leakage, severe bleeding, or organ failure). We evaluated the traditional and revised classification schemes against clinical intervention levels to determine how each captures disease severity using data from five years (2005–2010) of a hospital-based study of pediatric dengue in Managua, Nicaragua. Laboratory-confirmed dengue cases (n = 544) were categorized using both classification schemes and by level of care (I–III). Category I was out-patient care, Category II was in-patient care that did not meet criteria for Category III, which included ICU admission, ventilation, administration of inotropic drugs, or organ failure. Sensitivity and specificity to capture Category III care for DHF/DSS were 39.0% and 75.5%, respectively; sensitivity and specificity for SD were 92.1% and 78.5%, respectively. In this data set, DENV-2 was found to be significantly associated with DHF/DSS; however, this association was not observed with the revised classification. Among dengue-confirmed cases, the revised WHO classification for severe dengue appears to have higher sensitivity and specificity to identify cases in need of heightened care, although it is no longer as specific for a particular pathogenic entity as was the traditional schema.

## Author Summary

Dengue is a mosquito-transmitted viral disease that is a major public health problem worldwide. Dengue virus (DENV) infection leads to Dengue Fever (DF) and a spectrum of life-threatening syndromes with fluid loss and hypotensive shock or other severe manifestations. Recently, the traditional World Health Organization (WHO) dengue classification scheme (classic DF, Dengue Hemorrhagic Fever (DHF), and Dengue Shock Syndrome (DSS)) was replaced with Dengue without Warning Signs, Dengue with Warning Signs and Severe Dengue (SD). Using data from 544 laboratory-confirmed dengue cases recruited over five years of a hospital-based study of pediatric dengue in Managua, Nicaragua, we evaluated the traditional and revised classification schemes against clinical intervention levels (I–III) to determine how each captures disease severity. The sensitivity and specificity to capture Category III care for DHF/DSS were 39.0% and 75.5%, respectively, and for SD were 92.1% and 78.5%, respectively. Interestingly, DENV-2 was significantly associated with DHF/DSS; however, this association was not observed with the revised classification. This study indicates that among dengue-confirmed cases, the revised WHO classification appears to have higher sensitivity and specificity for identifying cases in need of heightened care, although it is no longer as specific for a particular pathogenic entity as was the traditional schema.

## Introduction

Dengue is an arthropod-borne viral disease with increasing prevalence in the last three decades, resulting in serious public health problems worldwide. With no vaccine or specific treatment to mitigate the natural history of the disease, a tool that can help clinicians detect and provide timely treatment is of utmost importance. The traditional World Health Organization (WHO) classification for dengue was implemented since 1974, based on experience with pediatric dengue in Thailand, and was then revised in 1997 [1], [2]. It classifies dengue disease as Dengue Fever (DF), Dengue Hemorrhagic Fever (DHF), and Dengue Shock Syndrome (DSS). The case definition for DHF lists the presence of four criteria: fever, hemorrhagic manifestations, thrombocytopenia (platelets ≤100,000 cells/mm^3^), and evidence of plasma leakage (pleural effusion, ascites, hemoconcentration ≥20% or hypoproteinemia). In turn, DHF is divided into four grades (DHF I–IV), where Grades III and IV are DSS, with hypotensive shock or narrow pulse pressure plus clinical signs of shock. It has proved to be very useful, with an emphasis on volume replacement for hemodynamic instability. However, limitations have been noted regarding its complexity and applicability, particularly in patients with severe symptoms [3], [4], [5], [6], [7], [8]. The recognition of these limitations led the Tropical Disease Reseach branch (TDR) of the WHO in 2006–7 to sponsor a multicenter study in seven countries in Asia and Latin America [9], and from this study emerged a new classification schema [10]. The new classification is divided into Dengue without Warning Signs, Dengue with Warning Signs, and Severe Dengue. In this study, we evaluated the capacity of the traditional classification and the revised classification to detect severe cases of dengue, compared to standardized clinical intervention levels. This evaluation was performed with information from ∼550 laboratory-confirmed dengue patients 6 months to 14 years old seen at the National Pediatric Reference Hospital in Managua, Nicaragua, from 2005 to 2010.

## Materials and Methods

### Study Site and Population

A cross-sectional study was performed in the Hospital Infantil Manuel de Jesús Rivera (HIMJR), the National Pediatric Reference Hospital, in Managua, Nicaragua. A total of 544 children who attended the HIMJR between July 2005 and January 2010 with laboratory-confirmed dengue were studied. These patients were between 6 months and 14 years of age, had fever or history of fever less than 7 days, and one or more of the following signs and symptoms: headache, arthralgia, myalgia, retro-orbital pain, positive tourniquet test, petechiae, or signs of bleeding. Patients with a defined focus other than dengue were excluded. Additional exclusion criteria included: a) children weighing less than 8 kg, b) children less than 6 months of age, and c) children 6 years of age and older displaying signs of altered consciousness at the time of recruitment. Patient data such as vital signs, clinical data, and radiographic or ultrasound results were collected on a daily basis by trained medical personnel until discharge. A blood sample was collected daily for a minimum of three days for Complete Blood Count (CBC) with platelets, blood chemistry, and diagnostic tests for dengue. Between days 14 and 21 after onset of symptoms, a blood sample was taken for convalescent follow-up. Hospital admission criteria for study participants is detailed in Text S1. Criteria for admission to the Intensive Care Unit (ICU) included patients with shock despite appropriate fluid management with crystalloids and colloids, patients requiring vasoactive amines, patients using a mechanical ventilator, or patients requiring continuous monitoring due to hemodynamic instability. Over the years, a few patients were not able to be admitted to the ICU despite meeting ICU admission criteria due to the lack of space in the ICU.

### Ethics Statement

The protocol for this study was reviewed and approved by the Institutional Review Boards (IRB) of the University of California, Berkeley, and of the Nicaraguan Ministry of Health. Parents or legal guardians of all subjects provided written informed consent, and subjects 6 years of age and older provided assent.

### Data Management

All information was collected every 12 hours in Case Report Forms (CRFs) designed to follow the patients' progress. Each CRF was completed by an infectious disease pediatrician and supervised by a second physician. Following this supervision, the CRFs were systematically monitored and then their information was entered into an Access 2003 database by double-date entry, with quality control checks performed daily and weekly. Thus, all data were collected prospectively over the entire course of illness following the same protocol and were reviewed carefully in real time to minimize any missing data. The data were then analyzed by illness episode; there were no missing signs or symptoms by episode.

### Dengue Diagnosis

A case was considered positive for dengue when laboratory tests met one or more of the following criteria: 1) Dengue viral RNA was detected by RT-PCR, 2) Dengue virus (DENV) was isolated, 3) Seroconversion of DENV-specific IgM was detected by MAC-ELISA in paired acute and convalescent samples, and 4) DENV-specific antibody titer by Inhibition ELISA [11], [12], [13] demonstrated a 4-fold or greater increase between acute and convalescent sera. Primary DENV infections were those in which acute antibody titer was <10 or convalescent antibody titer was <2,560, and secondary infections were those in which antibody titer was ≥10 (acute) or ≥2,560 (convalescent) as determined by Inhibition ELISA.

### WHO Classifications

The traditional WHO classification is defined as follows: Dengue Fever (DF), Dengue Hemorrhagic Fever (DHF), and Dengue Shock Syndrome (DSS), whereas the revised WHO classification consists of Dengue without Warning Signs, Dengue with Warning Signs, and Severe Dengue (Table 1). Regarding the traditional classification, laboratory-confirmed cases that met the case definition for dengue but did not comply with the criteria for DHF or DSS were classified as DF. With respect to the revised classification, we interpreted SD to include compensated shock based on dengue case management algorithms in the 2009 WHO Guidelines [10]. A computerized algorithm was developed to classify laboratory-confirmed dengue patients according to the traditional and revised classifications; this algorithm compiled the presence or absence of all signs and/or symptoms as well as results of clinical laboratory tests and radiography/ultrasound and thereby determined the level of severity according to each of the classifications (Table S1).

**Table 1:** Traditional and revised WHO classifications for dengue severity [2], [10].

Previous WHO Classification for Dengue Severity
**Dengue Fever (DF)**Acute febrile illness with two or more of the following:
• Headache
• Retro-orbital pain
• Myalgia
• Leukopenia
• Arthralgia
• Rash
• Hemorrhagic manifestations
• Supportive serology or occurrence at the same location and time as other confirmed cases of dengue fever.
**Dengue Hemorrhagic Fever (DHF)**All of the following must be present:
• Fever or history of acute fever, lasting 2–7 days, occasionally biphasic.
• Hemorrhagic manifestations:
– Positive torniquet test;
– Petechia, equimosis, purpura or bleeding from mucosa, gastrointestinal tract, injection sites or other locations; or
– Haematemesis/melena.
• Thrombcytopenia (<100,000 platelets per mm^3^).
• Evidence of plasma leakage due to increased vascular permeability.
**Dengue Shock Syndrome (DSS)**DHF with hypotension for age or narrow pulse pressure (<20 mmHg), plus one of the following:
• Rapid and weak pulse
• Cold, clammy skin, restlessness

Revised WHO Classification for Dengue Severity
**Dengue without Warning Signs**Fever and two of the following:
• Nausea, vomiting
• Rash
• Aches and pains
• Leukopenia
• Positive torniquet test
**Dengue with Warning Signs**Dengue as defined above with any of the following:
• Abdominal pain or tenderness
• Persistent vomiting
• Clinical fluid accumulation
• Mucosal bleeding
• Lethargy, restlessness
• Liver enlargement >2 cm
• Laboratory: increase in HCT concurrent with rapid decrease in platelet count
**Severe Dengue**Dengue with at least one of the following criteria:
• Severe Plasma Leakage leading to:
– Shock (DSS)
– Fluid accumulation with respiratory distress
• Severe Bleeding as evaluated by clinician
• Severe organ involvement
– Liver: AST or ALT≥1000
– CNS: impaired consciousness
– Failure of heart and other organs

The following definitions were used for each of the warning signs: Abdominal pain: abdominal tenderness and continuous pain (not intermitent), on some occasions diffuse. Persistent vomiting: more than three episodes of vomiting in 12 hours, preventing adequate oral hydration.

Clinical accumulation of liquids: pleural effusion and ascites diagnosed clinically, confirmed with imaging techniques (ultrasound for ascites, gallbladder wall thickening, and pleural effusion, and/or X-rays for pleural effusion). Mucosal bleeding: bleeding gums or conjunctiva, epistaxis, vaginal bleeding, bleeding from digestive, respiratory or urinary system (kidneys); mucosa defined as respiratory, vaginal, digestive, conjunctival and urinary tract mucosa. Lethargy: evaluated as an alteration of consciousness with a Glasgow score less than 15 or a Blantyre score less than 5. Irritability: irritability or restlessness. Hepatomegaly: the liver edge palpated by the clinician more than 2 cm below the costal margin. Increased hematocrit concurrent with rapid decrease in platelet count: increase in hematocrit together with a decrease of >10,000 platelets/mm^3^ in 24 hours with respect to previous measurement or concurrent with platelet count ≤100,000 cells/mm^3^.

### Statistical Analysis

The level of agreement for detection of severe cases of dengue between the traditional and revised classifications was determined using the Kappa index, as was the concordance between the clinical diagnosis by the physicians and the diagnosis generated by the computer algorithm. A Kappa value <0.00 was considered “poor agreement,” 0.00–0.20 as “slight agreement,” 0.21–0.40 as “fair agreement,” 0.41–0.60 as “moderate agreement,” 0.61–0.80 as “substantial agreement,” and 0.81–0.99 as “almost perfect agreement” [14]. To determine the sensitivity, specificity, and positive and negative predictive values of the traditional and revised WHO classifications for the detection of severe cases of dengue, each classification schema was compared to standardized clinical intervention levels. Three reference levels (gold standard) were established based on the type of intervention the patient required in accordance with the DENCO study sponsored by TDR [9]. *Category I* were those patients who were managed as outpatients (did not present criteria for hospitalization). *Category II* were hospitalized patients who received intravenous fluids for rehydration or maintanance and did not suffer organ damage. *Category III* were patients hospitalized in the Intensive Care Unit (ICU), administered inotropic drugs or ventilation, or who experienced organ failure. Patients classified as DHF or DSS were considered severe, and those classified as DF were considered non-severe. In the case of the revised WHO classification, patients classified as Severe Dengue were considered severe, and those with Dengue with or without Warning Signs were considered non-severe. With respect to the reference levels, severe dengue cases were considered as patients managed with *Category III* care. All data was stored in Microsoft Office Access version 2003 and analyzed using Stata Intercooled 9.0 (StataCorp LP, College Station, Texas), with a 95% confidence level.

## Results

Of 901 suspected dengue cases, 544 (60.4%) were laboratory-confirmed as positive. Within these 544 laboratory-confirmed dengue cases, sex was found to be evenly distributed (50% female, 50% male). Age varied between 6 months and 14 years, with a median of 8.5 years (IQR 5.1–11.2 years), and 40.6% between 5 and 9 years of age. Immune response was determined in 525 patients, of which 309 (58.9%) presented a secondary immune response. DENV serotype was identified in 494 (90.8%) patients, with DENV-3 predominating in 287 (58.1%) of the cases, followed by DENV-2 in 161 (32.6%) and DENV-1 in 45 (9.1%) (Table 2).

**Table 2:** Demographic characteristics of study population, 2005–2010.

Demographic Characteristics	N (%)
**Suspected dengue**	901
Laboratory-confirmed dengue	544 (60.4)
Other febrile illness	357 (39.6)
**Age** a	
<1 year	19 (3.5)
1 to 4 years	110 (22.2)
5 to 9 years	221 (40.6)
10 to 14 years	194 (35.7)
**Sex** a	
Female	272 (50)
Male	272 (50)
**Immune response** a	
Primary	216 (41.1)
Secondary	309 (58.9)
**Dengue serotype** a	
DENV-1	45 (9.1)
DENV-2	161 (32.6)
DENV-3	287 (58.1)
DENV-3 & DENV-4	1 (0.2)

a Laboratory-confirmed cases.

### Evaluation of the Classifications

**Figure 1:**
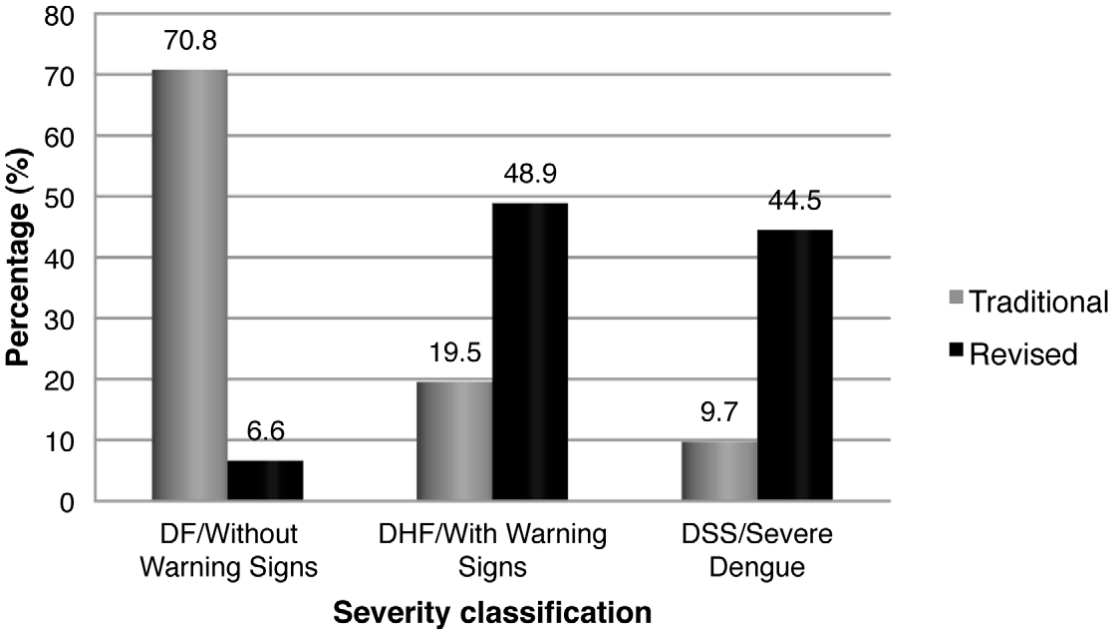
Traditional and revised WHO classification for dengue severity in Nicaraguan study, 2005–2010. The percentage of laboratory-confirmed dengue cases classified as DF (n = 385), DHF (n = 106), or DSS (n = 53) in the traditional scheme or classified as Dengue without Warning Signs (n = 36), Dengue with Warning Signs (n = 266), or Severe Dengue (n = 242) according to the revised scheme is shown.

According to the traditional WHO classification, the majority of the patients were classified as DF (385; 70.8%), while the remaining 29.2% were divided between DHF (106; 19.5%) and DSS (53; 9.7%). In contrast, with the revised scheme, although the majority of cases were classified as Dengue with Warning Signs (266; 48.9%), a large percentage of patients were classified as Severe Dengue (242; 44.5%) and only a small percentage (36; 6.6%) were classified as Dengue without Warning Signs (Figure 1). The level of agreement between the traditional and revised classifications for the detection of severe cases of dengue was fair (kappa 0.25, CI_95%_ 0.17–0.32, p<0.001) (Table 3), and the percentage of observed agreement (64.1%) was somewhat higher than that expected by chance alone (52.3%).

**Table 3:** Concordance^a^ between Traditional and Revised WHO classification in capturing severe cases of dengue, 2005–2010.

Traditional Classification	Revised Classification		Total
	Dengue with/without warning signs	Severe Dengue	
DF	246	139	385
DHF/DSS	56	103	159
Total	302	242	544

a Kappa = 0.25, CI_95%_ 0.17–0.32, p<0.001.

In the traditional classification, the majority of DF cases were treated at *Category II*, as they were hospitalized and received some type of intravenous (IV) rehydration. However, it is striking that 28.1% (108/385) of these patients received *Category III* intervention, as, despite being classified as DF, they showed severe clinical manifestations warranting ICU transfer or administration of inotropic drugs (Table 4). Ninety percent (48/53) of DSS cases were managed according to *Category III* care. However, most DHF cases (76.4%; 81/106) were treated at *Category II* care, and as such, sensitivity for detecting severe cases of dengue (DHF/DSS) was low (39.0%, CI_95%_ 31.8–46.6) and specificity was moderate (75.5%, 70.7–79.8) (Table 4).

**Table 4:** Traditional WHO classification of severity versus level of care, 2005–2010.

Traditional Classification	Level of care			Total
	Category I	Category II	Category III	
DF	83	194	108	385
DHF	4	81	21	106
DSS	0	5	48	53
Total	87	280	177	544

Sensitivity	Specificity	Positive predictive value	Negative predictive value
**39.0 (31.8–46.6)**	**75.5 (70.7–79.8)**	**43.4 (35.6–51.5)**	**71.9 (67.2–76.4)**

When the revised classification was compared with level of care (as the gold standard), 61.1% (22/36) of patients with Dengue without Warning Signs were treated as outpatients (*Category I*). In 38.9% (14/36) of remaining cases, 36.1% (13/36) fell under *Category II* care, and only one patient classified as Dengue without Warning Signs received *Category III* care. Sixty-seven percent (163/242) of Severe Dengue cases corresponded to *Category III* care, although it is noteworthy that 8 children (3.3%) of those classified as Severe Dengue were treated as outpatients (*Category I*). The fact that Severe Dengue cases mostly fall under *Category III* and non-severe cases of dengue (Dengue with and without Warning Signs) are categorized as *Category I* and *II* allows for a high level of sensitivity (92.1%, 87.1–95.6) and a moderate level of specificity (78.5%, 73.9–82.6) for the detection of severe dengue cases (Table 5). In this sense, the revised classification is more sensitive than the traditional classification, but equally specific.

**Table 5:** Revised WHO classification of severity versus level of care, 2005–2010.

RevisedClassification	Level of care			Total
	Category I	Category II	Category III	
Dengue without Warning Signs	22	13	1	36
Dengue with Warning Signs	57	196	13	266
Severe Dengue	8	71	163	242
Total	87	280	177	544

Sensitivity	Specificity	Positive predictive value	Negative predictive value
**92.1 (87.1–95.6)**	**78.5 (73.9–82.6)**	**67.4 (61.1–73.2)**	**95.4 (92.3–97.4)**

### Evaluation of Clinical Diagnosis

In 2009, the concordance between the clinician's diagnosis and classification according to the algorithms was evaluated prospectively (n = 212). With respect to the traditional classification, physicians had no difficulty classifying patients with DF or DSS, which matched the diagnosis generated by the computer algorithm in 95.6% (174/182), and 83.3% (5/6) of cases, respectively. Difficulty was encountered in classifying DHF patients, however, where only 12.5% (3/24) of cases were classified correctly, with 66.7% (16/24) being diagnosed as DSS by physicians. This incongruence meant that the level of agreement between clinical diagnosis and the computerized classification algorithm was moderate (kappa = 0.46, 0.38–0.55, p<0.001) (Table 6), with an observed agreement (85.8%) higher than expected by chance (73.6%).

**Table 6:** Concordance^a^ between clinical diagnosis and diagnosis by computer algorithm according to the traditional WHO classification.

Traditional WHO ClassificationClinical Diagnosis	Algorithm			Total
	DF	DHF	DSS	
DF	174	5	1	180
DHF	3	3	0	6
DSS	5	16	5	26
Total	182	24	6	212

a Kappa = 0.46, CI_95%_ = 0.38–0.55, p<0.001.

When comparing the physicians' clinical diagnosis with the computer-generated algorithm of the revised WHO classification, 72.2% (13/18) of patients with Dengue without Warning Signs and 87.8% (94/107) of patients with Severe Dengue were correctly classified. A lower percentage of cases were correctly diagnosed in those patients with Dengue with Warning Signs, where 66.7% (58/87) of cases were correctly classified. The level of agreement was substantial (kappa = 0.62, 0.53–0.71, p<0.001) (Table 7), with the observed agreement (77.8%) much higher than the expected (41.3%). In general, it was found that physicians had fewer difficulties classifying patients when using the revised classification scheme.

**Table 7:** Concordance^a^ between clinical diagnosis and diagonsis by computer algorithm according to the revised WHO classification.

Revised WHOClassificationClinical Diagnosis	Algorithm			Total
	Dengue without Warning Signs	Dengue with Warning Signs	SevereDengue	
Dengue without Warning Signs	13	15	2	30
Dengue with Warning signs	3	58	11	72
Severe Dengue	2	14	94	110
Total	18	87	107	212

a Kappa = 0.62, CI_95%_ = 0.53–0.71, p<0.001.

During the study period, DENV-1, DENV-2, and DENV-3 circulated among the patients. Association of disease severity with serotype was investigated using the two classification schemes. Using the traditional WHO classification protocol, it was found that the proportion of DHF and DSS cases was significantly greater (p<0.001, Fisher's exact test) in patients with DENV-2 infections as compared to the other serotypes (28.6% DHF and 22.3% DSS in DENV-2 infections versus 15.6% DHF and 11.1% DSS in DENV-1 infections and 16.4% DHF and 3.8% DSS in DENV-3 infections) (Table 8). Likewise, DENV-2 was most associated with evidence of plasma leakage, such as ascites, pleural effusion, and gallbladder wall thickening (p<0.001), as well as thrombocytopenia (p<0.001) (Table S2). In contrast, no significant difference between the proportion of severe cases and serotype was observed when the revised classification was applied (p = 0.104, Fisher's exact test), with 51.1%, 52.8% and 40.8% of severe dengue in patients with DENV-1, DENV-2, and DENV-3 infections, respectively (Table 8).

**Table 8:** Association between traditional and revised WHO classifications for dengue severity and serotype, 2005–2010.

WHO Classification	Serotype				p-value^a^
	DENV-1N = 45N (%)	DENV-2N = 161N (%)	DENV-3N = 287N (%)	DENV-3 & DENV-4N = 1N (%)	
**Traditional** b					
DF	33 (73.3)	79 (49.1)	229 (79.8)	1 (100)	<0.001
DHF	7 (15.6)	46 (28.6)	47 (16.4)	0	
DSS	5 (11.1)	36 (22.3)	11 (3.8)	0	
**Revised** b					
Dengue without Warning Signs	2 (4.5)	5 (3.1)	20 (7.0)	0	0.104
Dengue with Warning Signs	20 (44.4)	71 (44.1)	150 (52.2)	0	
Severe Dengue	23 (51.1)	85 (52.8)	117 (40.8)	1 (100)	

a p-value for Fisher's exact test.

b If the case of DENV-3 & DENV-4 is excluded, the p-value for Fisher's exact test for the traditional WHO classification is <0.001 and for the revised WHO classification, p = 0.087.

## Discussion

This study shows that the sensitivity and specificity of the traditional WHO classification for the detection of severe cases of dengue was 39.0% and 75.5%, respectively, while the sensitivity and specificity of the revised classification was 92.1% and 78.5%, respectively. A fair level of agreement (kappa = 0.25, p<0.001) was observed between the traditional and revised classifications for detection of severe cases of dengue. Evaluation of physicians' clinical diagnosis resulted in moderate agreement (kappa = 0.46, p<0.001) with the traditional classification and substantial agreement (kappa = 0.62, p<0.001) with the revised classification. However, whereas the traditional classification demonstrated a significant association of DENV-2 infection with DHF/DSS, no such association with Severe Dengue was observed with the revised classification scheme.

The traditional WHO classification includes two major entities, DF and DHF/DSS. This classification was largely based on experience with a pediatric population in Southeast Asia, though currently dengue has spread to other tropical and subtropical regions and clinical presentation of the disease has changed. Dengue varies widely in clinical manifestations, and the classification of severity therefore depends on the presence and detection of particular symptoms and signs. While the traditional classification requires the presence of four criteria to qualify as a case of DHF, situations have been observed where all four criteria are not present, resulting in problems with the classification and detection of severe cases. Indeed, many authors have reported difficulty in complying with the traditional classification for documenting clinical presentations of dengue such as hemorrhagic manifestations [4], [15], thrombocytopenia [15], [16], [17], [18], [19], and fluid leakage [4], [15], [20], [21], [22]. For instance, with respect to the latter, it is often difficult to demonstrate that hemoconcentration is ≥20%, as there are places where it is not possible to perform daily CBC; in addition, a physician-ordered intervention during the course of the illness, such as administration of intravenous fluids, can alter hematocrit levels and thus hemoconcentration [15]. Another complication is that few institutions in dengue-endemic countries have records of a normal hematocrit value for each patient; therefore, some investigators have used a population hematocrit value as a baseline or the hematocrit value during the convalescent phase or at discharge to define hemoconcentration via comparison with the highest hematocrit observed during the acute phase of the disease [4], [17]. Use of a population baseline enables increased documentation of plasma leakage, but may be less specific since DENV-negative cases can present with elevated hematocrit [17]; whereas using the convalescent hematocrit value as baseline requires retrospective classification of dengue cases.

Despite the widespread recognition of the usefulness of the traditional classification, difficulties in documenting all of the clinical manifestations required to define severe cases of dengue has resulted in alternative designations of certain clinical presentations seen in dengue, such as “Dengue with Signs Associated with Shock” (DSAS) [13] and “Dengue with Severe Bleeding” (DFB) [8]. As a result of this situation, Bandyopadhyay [6] proposed the creation of a multicentric prospective study in various countries of Asia and Latin America to describe the varying clinical presentations of dengue and to determine whether revision of the traditional WHO classification was necessary. Such a study was conducted from 2006–2007, and from it emerged a revised proposal for dengue classification [9]. Three levels of care were used in this study as the reference or gold standard and were based on the type of care needed and the condition in which patients presented. *Category III* represented patients with a severe condition and served as the comparison for DHF/DSS cases in the traditional classification and Severe Dengue cases in the revised classification. This methodology [9] was used as a basis for the study reported here.

In our study, 71% of patients were classified as DF, 19.5% as DHF, and only 9.7% as DSS according to the traditional classification, while only 6.6% of patients were classified as Dengue without Warning Signs, 48.9% as Dengue with Warning Signs, and 44.5% as Severe Dengue according to the revised classification. The difference in the percentage of severe cases between the two classification schemes can be explained by the existence of 62 patients with hypotension for age who did not present with platelet count ≤100,000, hemoconcentration or hemorrhagic manifestations, and were thus classified as DF, and 101 patients with compensated shock who were also classified as DF using the traditional scheme. These cases can serve to explain the low sensitivity (39.0%) of this classification scheme for detecting severe cases of dengue. The revised classification does not require the presence of the four criteria to determine severity, so the presence of shock, independent of thromobocytopenia or hemoconcentration, is sufficient for a case to be designated severe, and this explains the higher numbers of these cases within this group of patients.

The high sensitivity of the revised classification (92.1%) for detecting severe cases of dengue can be explained by the same reason, that is, the presence of a single criterion for defining a severe case. This feature allows better case capture and increased admission to health units, though it results in not all cases being truly “severe”, as expressed by a moderate positive predictive value (67.4%). This may overload health units in countries such as Nicaragua where large numbers of patients are admitted, disease evolution is carefully observed and monitored, and patients are discharged slowly once cases of severe disease have been ruled out. This over-estimation of severe cases of dengue may overwhelm hospitals and health centers, particularly during outbreaks or periods of high incidence, thus resulting in overextension of medical personnel and resources of each unit, but would avoid deaths due to the disease. In our pediatric cohort study of dengue in Nicaragua, during the years 2004–2008, the percentage of patients with dengue who were transferred to the study hospital from our study health center varied between 11% and 36%, but during 2009, the year where the revised WHO dengue classification scheme was implemented in the cohort study, the percentage of transferred cases rose to 83% (A. Balmaseda, G. Kuan, E. Harris, unpublished data). The revised classification has a specificity of 78.5% for detecting severe cases of dengue, which is virtually identical to that of the traditional classification (75.5%), with the exception that the revised classification scheme has a significantly higher negative predictive value. This feature of the revised classification may allow the clinician to better discern a patient who does not have a severe case of dengue.

From the treating physician's viewpoint, the revised classification may be useful because it allows the patient to be classified and treated in real-time, that is, during their hospital stay, whereas with the traditional classification scheme, the majority of cases tended to be retrospectively classified so as to detect the presence of the four criteria that define severity (DHF/DSS). This is reflected in the observed difficulty in correctly classifying the patient according to the traditional classification schema, expressed by the low level of agreement with the clinician's diagnosis (kappa = 0.46). Ideally, one would hope for a classification that is both sensitive and specific for detection of severe cases of dengue, in order to avoid oversaturation of health units, especially at the secondary level, but neither of the two classification schemes evaluated here possess these characteristics. Choosing a highly sensitive test maximizes the capture of severe cases, but requires subsequent evaluation during the patients' hospitalization to determine their real condition.

The traditional WHO classification allows characterization of the pathophysiology of severe cases of dengue as the syndrome of DHF/DSS. This focus on a particular syndrome is useful for investigating viral and immunological risk factors. For example, in this data set, DENV-2 was found to be significantly associated with DHF/DSS; this information is useful for clinicians and public health officials to keep in mind for future epidemics, as well as for designing possible follow-up investigations. However, this association was not observed when the new classification was used, presumably because the definition of severe dengue is so broad. DENV2 has been associated in this and numerous other studies with the defining features of DHF/DSS [23], [24] and with DHF/DSS to a greater extent than other serotypes [24], [25]. Therefore, it is not surprising that DENV2 is significantly associated with severity (DHF/DSS) using the previous classification scheme, but not with the revised classification scheme, which is no longer specific for DHF/DSS or the key clinical manifestations (e.g., thrombocytopenia, shock).

“Severe dengue” and “Dengue with warning signs” are very broad definitions that make it difficult to determine the pathophysiology of the disease. Therefore, it is important to analyze the frequency of warning signs and of severe manifestations, respectively, in order to obtain a clearer picture of the disease profile. Similarly, the specific syndrome of plasma leakage (DHF/DSS), so characteristic of the critical phase of dengue, is lost in the new classification; thus, for studies of viral, host, and immunological determinants of dengue pathogenesis, a more specific definition than “severe dengue” will need to be implemented. Additional implications of the new classification scheme exist with respect to epidemiological surveillance, since the traditional and revised categories are not equivalent and may initially lead to a difficult transition. According to results obtained in this study, we believe the revised classification is most useful for the physician for the detection of severe cases of dengue, but its utility in pathphysiological and epidemiological studies needs further evaluation in future research.

## Supporting Information

Table S1 **Summary of computerized algorithm to classify laboratory-confirmed dengue according to the traditional and revised classifications.**
(XLS)

Table S2 **Association between ultrasonographic and clinical laboratory results and DENV serotype, 2005–2010.**
(DOC)

Text S1 **Nicaraguan hospital-based DENGUE STUDY.**
(DOC)
