# Supplementary material for: Evaluation of the Traditional and Revised WHO Classifications of Dengue Disease Severity
Source: PLoS Negl Trop Dis. 2011 Nov 8;5(11):e1397. doi: 10.1371/journal.pntd.0001397 (PMC3210746; doi:10.1371/journal.pntd.0001397)
Supplement: Table S2 — Association between ultrasonographic and clinical laboratory results and DENV serotype, 2005–2010. (DOC) [file pntd.0001397.s002.doc]

**Table S2. Association between ultrasonographic and clinical laboratory results and DENV serotype,** 2005-2010.

| **Parameter** | **Serotype** | | | **p-value** |
| --- | --- | --- | --- | --- |
|  | DENV-1  N = 45  N (%) | DENV-2  N = 161  N (%) | DENV-3  N = 287  N (%) |  |
| Hepatomegaly^a^ | 36 (80) | 135 (84) | 242 (84) | 0.765^b^ |
| Splenomegaly^a^ | 5 (11) | 28 (17) | 45 (16) | 0.623^c^ |
| Ascites^a^ | 13 (29) | 83 (52) | 69 (24) | <0.001^b^ |
| Pleural effusion^a^ | 3 (7) | 55 (34) | 11 (4) | <0.001^c^ |
| Gall bladder wall thickening^a^ | 11 (24) | 87 (54) | 55 (19) | <0.001^b^ |
| Platelets <100,000/mm^3^ | 15 (33) | 103 (64) | 97 (34) | <0.001^b^ |
| WBC <4,000/mm^3^ | 40 (89) | 119 (74) | 248 (86) | 0.003^b^ |
|  |  |  |  |  |

^a^ Ultrasound results. Hepatomegaly: increase in liver size for patient age; splenomegaly: increase in spleen size for patient age; ascites: presence of liquid in the abdominal cavity; pleural effusion: presence of liquid >5 cc in the major pleural cavity; gall bladder wall thickening: thickening ≥3mm of the gall bladder wall.

^b^ p-value for Chi-square test.

^c^ p-value for Fisher´s exact test.
