# Supplementary material for: Evaluation of the Traditional and Revised WHO Classifications of Dengue Disease Severity
Source: PLoS Negl Trop Dis. 2011 Nov 8;5(11):e1397. doi: 10.1371/journal.pntd.0001397 (PMC3210746; doi:10.1371/journal.pntd.0001397)
Supplement: Text S1 — Nicaraguan hospital-based DENGUE STUDY. (DOC) [file pntd.0001397.s003.doc]

**Text S1.**

**Nicaraguan Hospital-based Dengue Study: Hospitalization Criteria prior to 2009**

- **Hospitalization Criteria for Suspected Dengue Cases**

1. Less than 1 year of age
2. Patients with DHF
3. Obesity
4. Non-tolerance for oral rehydration
5. Chronic illness (diabetes, asthma, etc.)
6. Dehydration
7. Signs of shock:
   - Hypothermia
   - Abundant perspiration
   - Cold, clammy skin
   - Exaggerated paleness
   - Oliguria
   - Tachycardia
   - Narrow pulse pressure (difference between systolic and diastolic <20 mm Hg)
   - Hypotension
8. Restlessness or weakness
9. Respiratory distress
10. Evidence of vascular leak

- Hemoconcentration
- Pleural effusion
- Ascites
- Oliguria

1. Presence of warning signs
   - **Warning Signs**
2. Intense and sustained abdominal pain
3. Abdominal distension
4. Thoracic pain
5. Postural (orthostatic) hypotension or fainting (syncope) (>5 years old)
6. Somnolence and irritability
7. Painful hepatomegaly
8. Frequent vomiting – non-tolerance for oral rehydration
9. Difficulty breathing (dyspnea)
10. Epistaxis, gingivorragia, hematemesis, melena. Presence of petechiae without other hemorrhagic manifestation does not require hospitalization.
11. Decreased urine production (oliguria)
12. Thrombocytopenia (platelet count <100,000/mm^3^)
13. Abrupt increase in hematocrit

**Nicaraguan Hospital-based Dengue Study: Hospitalization Criteria for 2009 and after**

Same as above except two additional Warning Signs were included:

1. Hepatomegaly (liver edge palpated >2 cm below the costal margin).

2. Increased hematocrit concurrent with rapid decrease in platelet count
